# Supplementary material for: Family support and prayer are invaluable coping strategies for our recovery: Experiences of persons living with cardiovascular diseases
Source: PLoS One. 2025 Jan 22;20(1):e0317314. doi: 10.1371/journal.pone.0317314 (PMC11753639; doi:10.1371/journal.pone.0317314)
Supplement: S1 Checklist — (DOC) [file pone.0317314.s001.doc]

**COMPLETED CONSOLIDATED CRITERIA FOR REPORTING QUALITATIVE STUDIES (COREQ): 32-ITEM CHECKLIST**

| **No** | **Item** | **Guide questions/description** |
| --- | --- | --- |
| **Domain 1:**  **Research team**  **and reflexivity** |  |  |
| Personal  Characteristics |  |  |
| 1. | Interviewer/facilitator | Which author/s conducted the interview or focus group?  **Answer:**  1. Frank Edwin,  2. Nancy Innocentia Ebu Enyan,  3. Bennett Owusu,  4. Nkosi Nkosi Botha,  5. Ivy Selorm Tsedze |
| 2. | Credentials | What were the researcher's credentials? *E.g. PhD, MD*  **Answer:**  1. Frank Edwin – Professor (Pro-Vice Chancellor)  2. Nancy Innocentia Ebu Enyan – Professor  3. Bennett Owusu – MD  4. Victor Kwasi Dumahasi – PhD Candidate  5. Nkosi Nkosi Botha – PhD Candidate  6. Ivy Selorm Tsedze – MPhil Candidate |
| 3. | Occupation | What was their occupation at the time of the study?  **Answer:**  1. Frank Edwin – Lecturer (Pro-Vice Chancellor) and Cardiologist  2. Nancy Innocentia Ebu Enyan –Lecturer (Dean of faculty)  3. Bennett Owusu – Cardiologist  4. Victor Kwasi Dumahasi – PhD Candidate  5. Nkosi Nkosi Botha – PhD Candidate  6. Ivy Selorm Tsedze – MPhil Candidate and Cardio Nurse |
| 4. | Gender | Was the researcher male or female?  **Answer:** Four males and two females. |
| 5. | Experience and training | What experience or training did the researcher have?  **Answer:**  1. Frank Edwin – Over 10years as researcher and  reviewer  2. Nancy Innocentia Ebu Enyan – Over 10years as researcher and reviewer  3. Bennett Owusu – 3years as researcher  4. Victor Kwasi Dumahasi – 1year as researcher  5. Nkosi Nkosi Botha – 3years as researcher and  reviewer  6. Ivy Selorm Tsedze – 1year as researcher |
| Relationship  with  participants |  |  |
| 6. | Relationship established | Was a relationship established prior to study commencement?  **Answer:** Yes, three of the authors (Frank Edwin, Bennett Owusu, & Ivy Selorm Tsedze) are staff of the Cardio Centre and had provided care to the participants. |
| 7. | Participant knowledge of the interviewer | What did the participants know about the researcher? e*.g. personal goals, reasons for doing the research*  **Answer:** Researchers interested in the real-life experiences of participants to help inform policy and practice. |
| 8. | Interviewer characteristics | What characteristics were reported about the interviewer/facilitator? e.g. *Bias, assumptions, reasons and interests in the research topic*  **Answer:** None |
| **Domain 2:**  **study**  **design** |  |  |
| Theoretical  framework |  |  |
| 9. | Methodological orientation and Theory | What methodological orientation was stated to underpin the study? *e.g. grounded theory, discourse analysis, ethnography, phenomenology, content analysis*  **Answer:** Phenomenology |
| Participant  selection |  |  |
| 10. | Sampling | How were participants selected? *e.g. purposive, convenience, consecutive, snowball*  **Answer:** Purposive |
| 11. | Method of approach | How were participants approached? e*.g. face-to-face, telephone, mail, email*  **Answer:** Face-to-face. |
| 12. | Sample size | How many participants were in the study?  **Answer:** 17 |
| 13. | Non-participation | How many people refused to participate or dropped out? Reasons?  **Answer:** Five, not available during the study period. |
| Setting |  |  |
| 14. | Setting of data collection | Where was the data collected? e*.g. home, clinic, workplace*  **Answer:** Hospital (13 participants) and home (4 participants) |
| 15. | Presence of non-participants | Was anyone else present besides the participants and researchers?  **Answer:** No |
| 16. | Description of sample | What are the important characteristics of the sample? *e.g. demographic data, date*  **Answer:** Gender, age, level of education, and occupation |
| Data collection |  |  |
| 17. | Interview guide | Were questions, prompts, guides provided by the authors? Was it pilot tested?  **Answer:** Yes, questions, prompts, guides provided during interview. Instrument pilot tested. |
| 18. | Repeat interviews | Were repeat interviews carried out? If yes, how many?  **Answer:** Yes, follow-up interviews conducted during member-checking. |
| 19. | Audio/visual recording | Did the research use audio or visual recording to collect the data?  **Answer:** Audio recorders used during data collection |
| 20. | Field notes | Were field notes made during and/or after the interview or focus group?  **Answer:** Yes, field notes taken during and after interview. |
| 21. | Duration | What was the duration of the interviews or focus group?  **Answer:** 30-45mns |
| 22. | Data saturation | Was data saturation discussed?  **Answer:** Yes |
| 23. | Transcripts returned | Were transcripts returned to participants for comment and/or correction?  **Answer:** Yes, member-checking conducted |
| **Domain 3: analysis and findings** |  |  |
| Data analysis |  |  |
| 24. | Number of data coders | How many data coders coded the data?  **Answer:**  Four authors (Frank Edwin, Nancy Innocentia Ebu Enyan, Victor Kwasi Dumahasi, & Nkosi Nkosi Botha) conducted data coding, and two authors (Bennett Owusu & Ivy Selorm Tsedze) verified the codes. |
| 25. | Description of the coding tree | Did authors provide a description of the coding tree?  **Answer:** No, rather coding book was used. |
| 26. | Derivation of themes | Were themes identified in advance or derived from the data?  **Answer:** Theme emerged from data. |
| 27. | Software | What software, if applicable, was used to manage the data?  **Answer:** Qualitative data analysis software, NVivo version 14 |
| 28. | Participant checking | Did participants provide feedback on the findings?  **Answer:** Yes, were very pleased with findings. |
| Reporting |  |  |
| 29. | Quotations presented | Were participant quotations presented to illustrate the themes/findings? Was each quotation identified? e*.g. participant number*  **Answer:** Yes |
| 30. | Data and findings consistent | Was there consistency between the data presented and the findings?  **Answer:** Yes |
| 31. | Clarity of major themes | Were major themes clearly presented in the findings?  **Answer:** Yes |
| 32. | Clarity of minor themes | Is there a description of diverse cases or discussion of minor themes?  **Answer:** Yes |
